# Inadequacies in uveitis: misnomers, incongruencies, persistence of obsolete terminologies & inappropriate guidelines, treatment inadequacies, and misinterpretations

**DOI:** 10.1186/s12348-025-00470-0

**Published:** 2025-03-06

**Authors:** Carl P. Herbort, Ioannis Papasavvas, Masaru Takeuchi, Yoshihiko Usui, De-Kuang Hwang, Sagnik Sen

**Affiliations:** 1 Inflammatory and Retinal Eye Diseases, Centre for Ophthalmic Specialised Care (COS), 6 Rue Charles-Monnard, Lausanne, 1003 Switzerland; 2 Department of Ophthalmology, National Defence Medical College, Tokorozawa, Saitama Japan; 3 Department of Ophthalmology, Tokyo Medical University, Tokyo, Japan; 4 Department of Ophthalmology, Taipei Veterans General Hospital, Taipei, Taiwan & School of Medicine, National Yang Ming Chiao Tung University, Taipei, Taiwan; 5 Medical Retina and Uveitis, Moorfields Eye Hospital, London, UK; 6 Vitreoretina, Guy’s and St Thomas’ Hospital, London, UK; 7 Uveitis and Scleritis Department, Moorfield’s Eye Hospital, London, UK

## Abstract

**Background:**

Inadequacies in medicine are manifold including inadequate influence of opinion leaders and consensus groups on terminology, diagnostic criteria and treatment guidelines, obsolete classifications and terms as well as misinterpretations of disease mechanisms. This is no different for uveitis and possibly even more pronounced as these are rare entities.

**Purpose:**

To underline inadequacies in uveitis including inadequate diagnostic criteria and treatment guidelines, misnomers, obsolescence of terminology, misinterpretation of disease processes and inadequate or underuse of investigative modalities in uveitis. This is a first report to be followed by others.

**Methods:**

A critical retrospective literature review of selected inadequacies in uveitis practice.

**Results:**

We investigate the mechanism of abuse of power of opinion leaders through the historical events such as the delay in acceptance of antiviral treatment for zoster ophthalmicus, report inadequacies and misnomers resulting from opinion articles or opinion surveys, inadequate treatment guidelines such as for Vogt-Koyanagi-Harada disease (VKH) , delays in adopting appropriate classifications, inappropriate pathophysiological interpretations such as for multiple evanescent white dot syndromes (MEWDS), reluctance to implement ICGA use, a crucial biomarker for choroiditis, among others.

**Conclusion:**

Inadequacies in uveitis are not so rare and often result from inadequate influence of opinion leaders oe groups. Some are harmless although annoying, such as misnomers, while others can be harmful such as inadequate treatment guidelines.

## Introduction

**Fig. 1:**
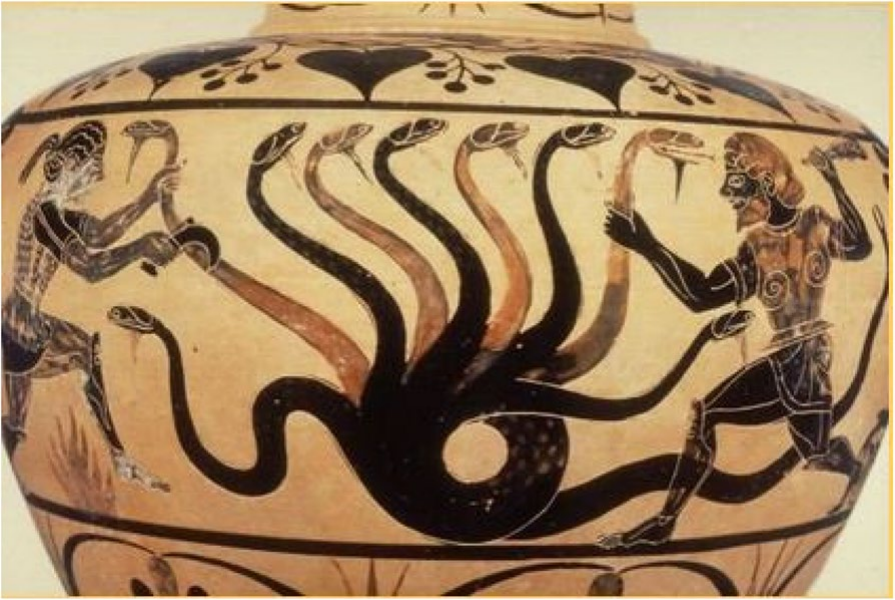
Heracles and his nephew Iolaos trying to kill the Hydra of Lerna as imaged in a Greek pottery. These are some introductory glimpses giving an idea of the scope of the work presented in this first article, addressing only limited selected issues. Topics will be presented randomly with tugs indicating the type of inadequacy(ies) applying to each topic

Inadequacies in medicine resemble the Hydra of Lerna, a fire-breathing monster with multiple serpent heads. When one head was cut off, two would grow in its place. Its killing was one of the twelve works of Heracles in which he was finally successful with the help of his nephew (Fig. 1). In our daily practice it is not so rare to come across inadequacies, an occurrence which seems impossible to eliminate as more come out successively. Some of them are harmless although annoying, created by the need for pseudo-scientific innovation. The use of cyanescence in Indocyanine Green angiography (ICGA) instead of fluorescence is an example. Others can affect the evolution of a patient’s eye problem such as the persisting inadequate treatment recommendations for acute posterior multifocal placoid pigment epitheliopathy (APMPPE). Others are obsolete terminologies, definitions and guidelines remaining in use due to medical community’s slow pace of accepting change and integrating evolution in medical practice. For instance, for more than 20 years there have been attempts to abolish the unfounded and useless term “white dot syndromes” (WDS), painstakingly rolling the WDS stone up the hill, like Sisyphus, just to see the stone rolling down on the other side, with this inadequate misnomer devoid of sense being used over and over again. Indeed, once inadequate terms and denominations are ingrained in regular use, it is difficult and takes time to replace them with adequate terminologies. Often inadequate diagnostic criteria have been generated and disseminated and encrusted in the literature, often resulting from consensus groups/meetings not devoid of biases. The enigma is the rapidity with which these unfounded notions have been quickly adopted at large and repeated without sound questioning.

### First contact with inadequacies: the herpes zoster ophthalmicus case (delay and reluctance in accepting of antiviral treatment)

In 1984, we started a double masked trial comparing 7 days versus 14 days of oral acyclovir treatment (ACV, 800 mg 5X/day) in acute herpes zoster ophthalmicus (AHZO), treated within 3 days of the occurrence of the cutaneous rash. Abstention of corticosteroid treatment (local or systemic) was part of the protocol, unless absolutely necessary. At that time systemic corticosteroids were the standard of therapy and we had a hard time to convince our hierarchy and the ethic committee to allow the study. In November 1985, Cobo & Coll. published an interim report on a placebo-controlled trial on the treatment of AHZO with ACV (600 mg/day for 10 days) within 7 days after the rash [1] with their final report out in June 1986, showing that the ACV treatment significantly reduced the incidence and severity of the most common complications of AHZO [2]. In October 1986, we published our preliminary results showing a drastic decrease of post-herpetic complications in both the 7-day and the14-day treatment groups [3], with our final results, including 48 patients published in 1991, showing that the severe long-term complications were reduced from 21% in a historical control group to 4% after a follow-up of at least 2 years [4]. Corticosteroid treatment had not been necessary in any of the patients. It is rare to obtain such an excellent therapeutic ratio as was the case for oral acyclovir in AHZO [4]. Since the completion of inclusion of our study (end of 1988) high-dose oral acyclovir became the standard of care for AHZO, given the spectacular results of our study and the equally unquestionable results of the study by Cobo & Coll. As late as 1991 authors from the Zoster Clinic of a renowned centre, published a double-masked trial of topical (!) acyclovir and steroids in the treatment of herpes zoster ocular inflammation, saying that “combined steroid and ACV was questionably better than steroids alone, causing marginally fewer rebound inflammation” [5]. We sent a letter to question these conclusions, exposing our results as well as citing the study of Cobo & Coll., showing without any doubt the excellent efficacy of ACV, adding that “on an ethical basis, no patient should be denied ACV treatment” [6] The response was: “as far as the efficacy of ACV is concerned, we are aware at present of only one controlled trial… and we feel that it is necessary to have another controlled trial” ([6], reply). This position followed an earlier statement: “In acute herpes zoster… ACV’s general use should not be encouraged, especially that the evidence suggests that it does not affect postherpetic neuralgia” [7]. Further in 1993, five years after the potent effect of ACV was undoubtfully proven by two controlled adequate studies, the following statements were published: “We therefore feel that systemic corticosteroids are indicated very early in patients”, this statement being applied to a highly infectious viral disease [5]. Further, statements like “systemic antivirals have proved rather disappointing in zoster” and “we feel that before substantial funding is used to finance use of the drug routinely in zoster more clinical trials are essential” are possibly the reason why the ACV in zoster picked up late in the UK and deprived patients of an extremely efficacious reimbursed therapy for some years. The reason to report this historical case is not so much to insist on the correct treatment of HZO which was already used in the rest of the world at the time of this futile controversy but to describe the mechanism of abuse of power by opinion leaders based on bad faith, misuse of influence and easy access to publication, a behaviour that is not part of the past but can still occur nowadays.

### Inadequacies and misnomers resulting from opinion articles, opinion surveys and “so-called” consensus meetings [(HLA-A29 birdshot retinochoroiditis (BRC), white dot syndromes (WDS), Vogt-Koyanagi-Harada disease (VKH)]

#### The inadequacy of consensus meetings or similar

Opinion articles that give guidelines, opinion surveys and so-called consensus meetings resulting in recommendations can be harmful and hinder the progress of medicine [8]. The process of opinion surveys/consensus meetings must be debarked and analysed [9]. What is the purpose and conduct of such meetings beyond the obvious hope to make medicine progress? When analysing consensus conferences, the general profile of such meetings is always quite similar. A group of individuals interested in promoting better knowledge of a given disease decide to call up a workshop to determine new criteria and definitions of a disease. To ensure the success of the meeting, which typically lasts only one or two days, guidelines need to be established in advance. These guidelines are then discussed by invited international participants, many of whom may not be true experts in the field and might be inclined to align with the suggested approach by the expert majority. The problem with this approach is the fact that these opinion leaders might be at the origin of biased notions. Organizers often possess sufficient influence to ensure that the conclusions of these international consensus meetings are published in reputable, high-impact journals. As a result, a relatively small group of individuals, supported by international “ratifying” participation, is able to set standards which, when inadequate, can take years to correct. Of course, this does not apply to maximally evidence-based trustworthy guidelines published after a thorough literature search at their base and integrating a plurality of views.

Several examples have shown that the restrictive kind of approach uncovered hereabove, based on surveys, polls, consensus decisions or expression of opinions turns out often to be counterproductive. This is because they may lead to inappropriate or incorrect recommendations due to bias toward specific groups that convened the meetings. These biased recommendations can cause a step backwards rather than progress. Two examples can be cited here to illustrate how such approaches can be harmful, as they may generate inadequate and incorrect results which can then hold the entire medical community hostage, as they become the "official guidelines" [10].

#### HLA-A29 birdshot retinochoroiditis (BRC) following consensus generated inadequate diagnostic criteria

In 2006, a set of diagnostic criteria for HLA-A29 Birdshot retinochoroiditis (BRC) was published as a result of a “consensus workshop” [11]. These “research criteria” have since been revised and corrected because of the many shortcomings they contained [12]. Despite the fact that such information was already available at the time of the workshop [13], the criteria did not consider well-established objective and scientifically relevant investigative methods such as indocyanine green angiography (ICGA), an essential diagnostic tool and criterion for BRC showing subclinical involvement in a normal appearing fundus [14]. Depigmented “birdshot lesions” which are merely choroidal scars indicating advanced disease were given excessive importance. Birdshot fundus lesions are just a comforting finding when they are present in patients, as they are a sign of already established disease. However, birdshot lesions are not required for diagnosis, as diagnostic hypofluorescent dark dots (HDDs) on ICGA appear well before birdshot lesions [15].

In addition to ignoring ICGA, another significant development in the appraisal of BRC, namely the detection of the HLA-A29 antigen by polymerase chain reaction (PCR) technique was not taken into account. The result of an improved search for the presence of HLA-A29 antigen by PCR technique, already known at the time of the workshop [16], was not integrated and HLA-A29 was categorised as supportive but not as required. Yet we now know that HLA-A29 is not only a supportive but an essential and required criterion as the positivity rate is close to 100%, when testing via PCR [17].

Beside the neglect of available new essential objective techniques, the appraisal and interpretation of classical clinical findings were not satisfactory either. Thus, the importance of retinal vasculitis was largely minimised, considering it merely as a supportive element of BRC, when retinal involvement has been described as part of the clinical picture in the early publications [18–20]. and in most subsequent reports [21, 22], illustrating the crucial importance of this feature. Recently, the BRC vasculitis pattern was even considered as being diagnostic [23, 24].

Furthermore, there are some minor but important points to consider. Among them, keratic precipitates (KPs) were incorrectly listed as an exclusion criterion. This is misleading because granulomatous KPs are seen in almost 20% of treatment-naive patients [25] and were already reported by Gass [19].

These incorrect criteria have hindered the accurate assessment of BRC for many years. Despite attempts to correct them [12], they continue to be cited repeatedly, highlighting the challenge of correcting inadequate and erroneous concepts, especially when published in reputable high-impact journals. They hindered the use of appropriate diagnostic criteria published since (Table 1).

**Table 1:**
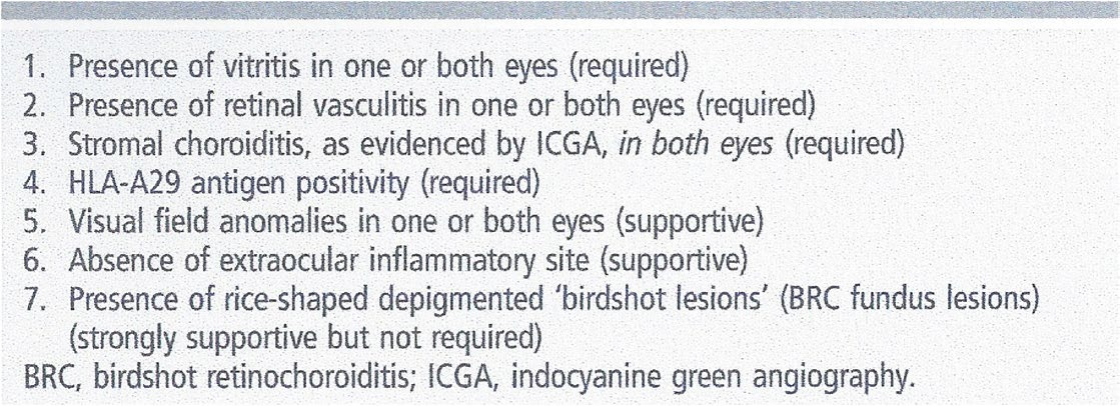
Clinically relevant diagnostic criteria for HLA-A29 birdshot retinochoroiditis (BRC) (Reprinted from Br J Ophthalmol. 2017 Jul;101(7):851-855)

Since then, the Standardization of Uveitis Nomenclature (SUN) Working Group including some authors of the first criteria have tried to correct their criteria using consensus-based selection and machine learning and finally added the crucial ICGA criteria [26]. However, the clinical use of the criteria is still unsatisfactory, as it was exclusively focused on choroidal disease when it is well-known that BRC is a dual retinal vasculitis and stromal choroiditis. For instance, the presence of retinal vasculitis is completely ignored in their revised criteria, although it is present in 100% of cases with a characteristic pattern and is usually the first sign seen in the absence of choroiditis. The retinal vasculitis is the source of the symptoms which push the patients to consult, i.e. poor vision, problems with peripheral vision, night vision flickering lights, all due to the oedema caused by vasculitis. Omitting this crucial criterium is weakening their criteria. They maintained from the first set of criteria the absence of KPs, their presence being an exclusion criterium while KPs were found to be present in up to 20% of cases. To find this sign, however, a sufficiently large series of non-treated cases is needed which can never occurs when cases are collected individually or in small numbers from different centres as was the case for the SUN attempt. A significant anterior uveitis requiring drops is however not expected. It shows also that citations taken into account by the group followed a biased choice deliberately ignoring work done by others in other parts of the world.

#### Inadequate diagnostic criteria and management following a consensus meeting on VKH

The limitations of consensus derived decisions apply also to the “revised VKH diagnostic criteria” published in 2001 as a result of another consensus workshop held in 1999 [27]. The criteria subdivided VKH into 3 categories depending on the proportion of positive clinical signs, namely complete, incomplete and probable forms of VKH disease. The incongruity of the system was the fact that it mixed acute and chronic signs that never occur together. For this reason, the “so-called” complete form of the disease could essentially not be diagnosed as it listed both acute and chronic signs. This system was not practically relevant nor clinically useful for managing the disease, but it led to many irrelevant and impossible studies showing that the complete form almost never occurred according to the proposed system [28, 29]. The criteria not only contributed little to the appraisal of VKH but overshadowed the real relevant approach of the disease, the crucial subdivision of VKH cases into acute and chronic forms and their relevant consequences on both treatment and disease prognosis [30]. Indeed, the appraisal of VKH should be based on the distinction of two forms of the disease, acute-onset VKH and chronically evolving VKH disease and separate diagnostic criteria apply to each form [31] (Table 2).

**Table 2:**
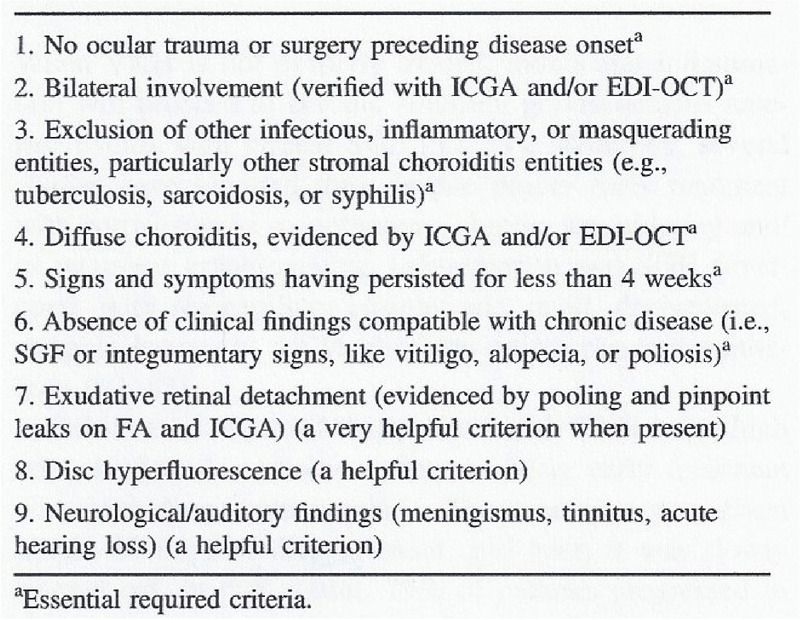
Clinically relevant and simplified diagnostic criteria for acute-onset VKH (Reprinted from Eye (Lond). 2022 Jan;36(1):29-43)

The importance of distinguishing the two forms resides in the fact that the response to treatment and outcome differ. If adequate treatment is introduced within the therapeutic window of opportunity [32], evolution to the chronic form can be avoided in a high proportion of cases and there is a chance to cure the disease [33]. SUN also tackled the diagnosis of VKH and marked significant progress over the revised diagnostic criteria of 2001, as it subdivided the disease into acute and chronic forms [34], as recommended by us and other groups [30, 32, 35, 36]. However, these criteria lack usefulness especially because the delineation between acute-onset and chronic disease has not been clearly defined and should correspond to less than 3-4 weeks, the therapeutic window of opportunity as defined by Abu El Asrar’s group [32]. This is a crucial point as already indicated, because if appropriate dual corticosteroid and immunosuppressive treatment is applied within this period, when anterior inflammation is still minimal, healing of the disease can be expected [33].

As illustrated by the inappropriate diagnostic criteria for BRC and VKH, the history of consensus-driven criteria demonstrates the limitations and potential pitfalls of relying on collective opinion rather than rigorous, evidence-based analysis which have become available in most cases. These experiences underscore the importance of a critical and informed disease classification and diagnosis approach.

#### Abandoning the use of the unfounded misnomer term “white dot syndromes” (WDS) is long overdue

The WDS term was created based on the mistaken belief that all diseases with the clinical sign of white fundal dots shared a common mechanism, the granuloma [37]. Although the terminology was not the result of a proper consensus workshop, it can be assimilated to such a process, as after the publication of this conjectural hypothesis in1995 in a high impact journal, it was unbelievably quickly accepted at large by the ophthalmological community. Indeed, this potpourri terminology included ill understood diseases at the time such as multiple evanescent white dot syndrome (MEWDS), acute posterior multifocal placoid pigment epitheliopathy (APMPPE), BRC and others for which clinicians were happy to have a putative framework. Again, this is another example of opinions without a proper foundation being favoured, while neglecting objective and precise investigative methods like ICGA, already available at the time of the publication of their article, that could have prevented misleading the ophthalmological community for over 25 years. This aberrant idea of grouping all white-spotted diseases in a single group began to be challenged as early as 2002 [38] thanks to the insight and imaging access to the choroidal compartment through ICGA. It became obvious that the potpourri of white dot syndromes had to be abandoned and replaced by the appropriate term of non-infectious choroiditis, subdivided on one side into diseases of the choroidal stroma, i.e. stromal choroiditis including BRC or Sympathetic Ophthalmia (SO) or VKH and on the other side, diseases of the choriocapillaris, choriocapillaritis including MEWDS, idiopathic multifocal choroiditis (MFC), APMPPE and serpiginous choroiditis (SC) [39]. The issue of the useless and clinically irrelevant terminology of WDS seemed to be definitively settled in 2022, when an article recommended to abandon this obsolete useless misnomer [40]. In 2024, the first ophthalmology textbook came out not mentioning WDS any longer in its uveitis section [41]. Therefore, the fact that the International Uveitis Study Group (IUSG), after an opinion survey completely centred on WDS, decided to abandon the term for the follow-up consensus meeting has to be strongly praised. Nevertheless, this remained a process in form of an imperfect survey/consensus approach, when, as explained hereabove, the question had been solved by objective, scientific and evidence-based methods. Participants of the survey were asked for their opinion on points on which many of them were unfamiliar with, knowing that ICGA is underused in some parts of the world. In the survey, ICGA was much less commonly used even by those who responded that they used multimodal imaging (MMI). Furthermore, the diseases included in the survey are so rare that many of the participants, even when allegedly skilled in the topic, were not in a position to knowingly answer which resulted in potentially arbitrary and hence responses difficult to interpret. One positive aspect of the survey was that a large majority estimated that the WDS term should be abandoned [42]. Such an enterprise represented again a step backwards in tackling medical questions when modern methodology was at hand. Fortunately, at least, this project avoided further contribute to propagate and perpetuate of an inadequate and useless terminology, which many painstakingly worked to get rid of, obscuring an existing objective and reasoned approach to this issue that was available as early as 2002 [38].

### Acute posterior multifocal placoid pigment epitheliopathy (APMPPE) (inadequate treatment guidelines prevail – APMPPE is a misnomer)

#### Inadequate unsatisfactory treatment guidelines prevail

APMPPE is a choriocapillaritis characterized by inflammatory choriocapillaris non-perfusion at the origin of ischemic lesions of the outer retina with predominant damage to the photoreceptors and, to a lesser extent, damage to the retinal pigmentary epithelium (RPE) cells [43]. The disease process is reversible, however ischemia-induced chorioretinal scars may develop with negative consequences on visual function. The general treatment guidelines that seem to prevail at large is abstention of therapy as advocated in textbooks: (1) “…there is no evidence that corticosteroid treatment affects the short-term or long-term outcome. In most cases the visual outcome is good without therapy” [44]; (2) “Most patients with APMPPE have a good prognosis and do not require therapy” [45]. This therapeutical attitude, namely no therapy, is repeated over and over in courses and presentations, but without any scientific evidence. Participants falsely integrate that APMPPE should not be treated.

Here is an example that shows the consequence of the “no treatment” approach.

**Fig. 2:**
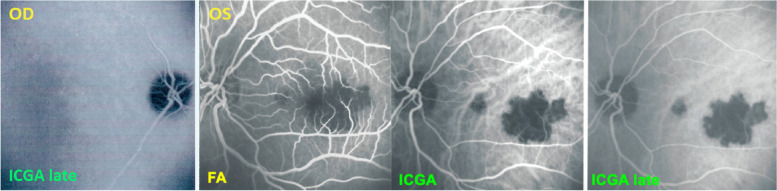
APMPPE/AMIC at first presentation elsewhere. FA OS early frame (second from left) shows central hypoperfusion, which corresponds to non-perfusion as shown on the two ICGA frames (right and second from right). The ICGA frame of OD shows discrete central hypoperfusion that announces ischemia of the second eye that occurred 3 weeks later

A 55-year-old man presented a febrile “viral” flu-like episode followed by a decrease of vision in his left eye with photopsias. The diagnosis of APMPPE was made elsewhere and it was decided not to introduce systemic treatment “as the disease is self-limited”. The FA/ICGA performed was compatible with APMPPE/AMIC (Fig. 2). If examined attentively, ICGA of the apparently normal right eye already showed central hypoperfusion.

Vision decreased further and after 3 weeks the same symptomatology of photopsias and visual field deficit was noted in the right eye. Preoccupied by the deleterious evolution of his first involved left eye, the patient decided to be seen in another hospital where the same diagnosis was made, namely APMPPE and again no treatment was introduced because of the allegedly self-limited character of APMPPE. With vision decreasing in both eyes the patient consulted our centre with a best corrected visual acuity (BCVA) of 0.05 OD and of 0.15 OS. Fundus examination showed a cicatricial macula on the left and confluent atrophic plaques on the right with some foci still active. ICGA showed macular hypofluorescence due to macular atrophy on the left and central hypofluorescence due to non-perfusion on the right surrounded by less hypofluorescent areas indicating still active lesions (Fig. 2).

**Fig. 3:**
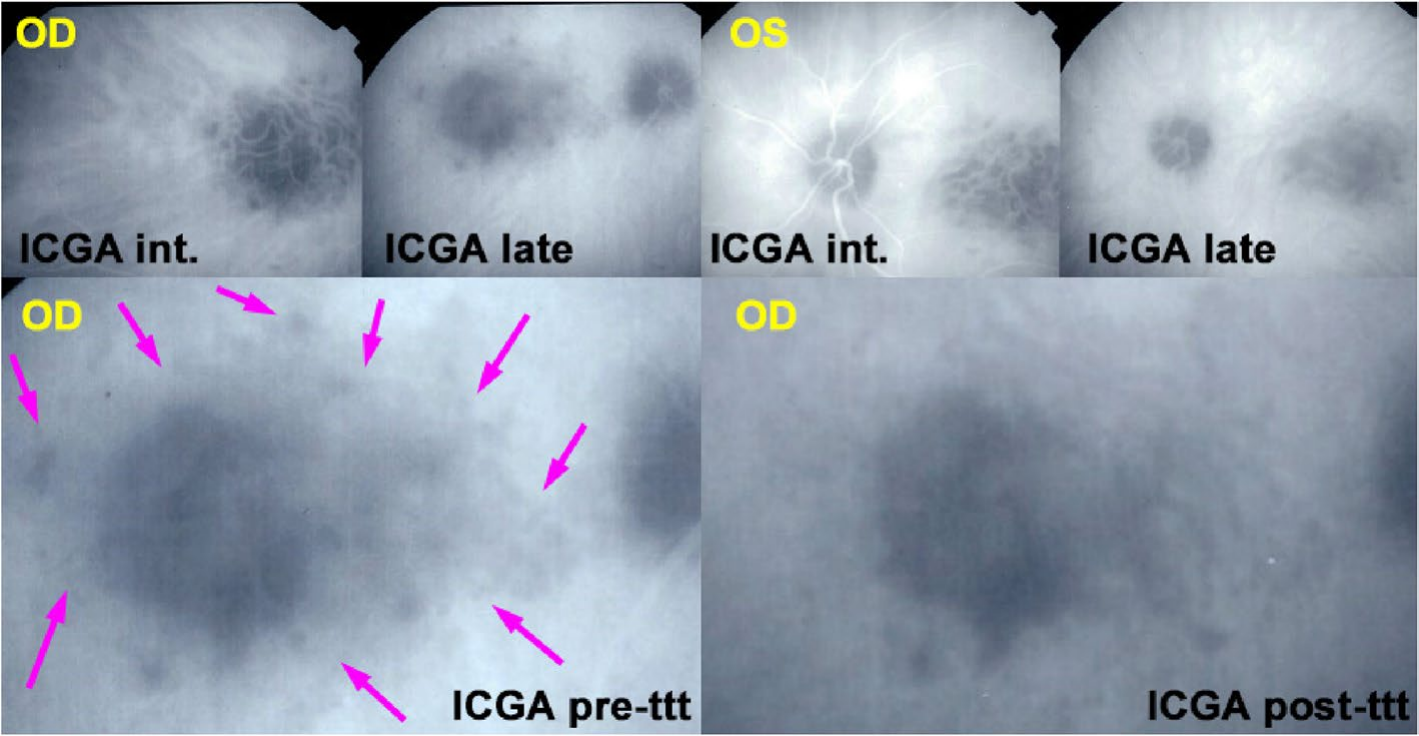
APMPPE/AMIC; ICGA in a case that did not receive prednisone therapy with evolution towards macular atrophy ODS: top frames show ICGA findings in intermediate and late phases in both eyes at presentation 3 months after onset of disease. The bottom left frame shows active perimacular lesions less hypofluorescent than the centre (crimson arrows) that responded to prednisone therapy with regression of perimacular lesions after treatment but persistence of centromacular atrophy (bottom right frame)

We immediately started corticosteroid therapy, giving 60 mg prednisone per day that allowed to reverse the peripheral lesions OD, the second involved eye (Fig. 3) with an increase of BCVA to 0.15 but no effect on the initially involved left eye where the situation was already cicatricial and atrophic. This case is an illustration on how dangerous it is to follow the textbooks recommending abstaining from treatment while close morphological and functional monitoring is essential and in case of doubt initiation of treatment is crucial considering the potentially good risk/benefit ratio.

Our teaching for APMPPE has always been not to hesitate to resort to systemic corticosteroid treatment if there is the slightest suspicion of deleterious evolution. Close follow-up of the morphological evolution by ICGA, Optical Coherence Tomography (OCT) and OCT-Angiography (OCT-A) and/or functional evolution using visual field testing and microperimetry should encourage prompt intervention if necessary. In a recent study, analyzing one of the largest APMPPE series, we showed that more than 2/3^rds^ of patients needed systemic corticosteroid treatment, combined with immunosuppressants in 2 patients [46]. Follow-up data showed that mean VA in non-treated eyes was significantly lower than in the group of treated eyes clearly showing that in eyes for which treatment was not judged necessary VA suffered, indicating that decision to treat was probably taken insufficiently often inferring that an even higher proportion of patients probably need treatment. APMPPE is the perfect example of guidelines perpetuated from one textbook to another concerning a disease for which large series are scarce and no evidence-based information allowed to give accurate recommendations. Nevertheless, common sense dictates that potentially cicatrizing inflammation should be submitted to treatment.

#### APMPPE is a misnomer of a disease that should more appropriately be called AMIC

**Fig. 4:**
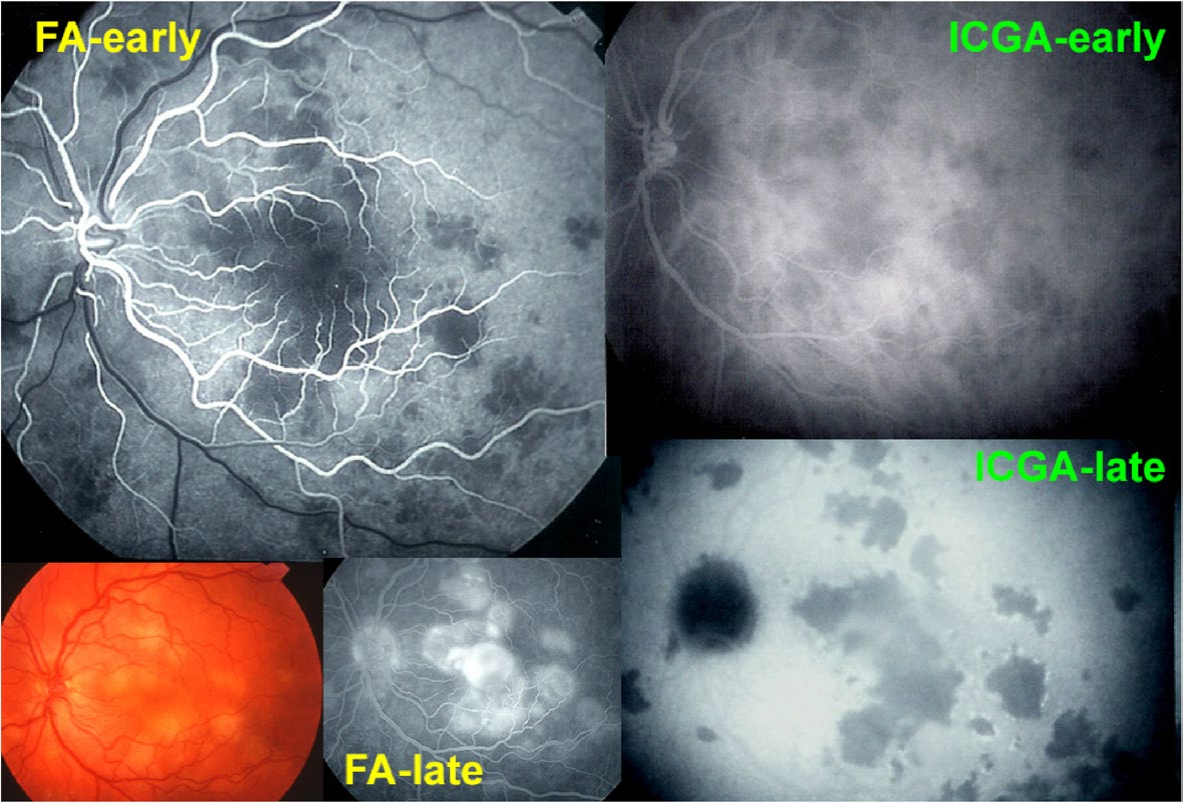
Case of APMPPE illustrating how Deutman proposed the correct physio-pathological explanation of APMPPE and renamed the disease acute multifocal ischemic choriocapillaritis AMIC). The choriocapillaris is accessible to FA in the first 60’’ of angiography and early perfusion delay on FA was interpreted by Deutman as choriocapillaris non-perfusion, a fact that was confirmed later by ICGA as the drop-out areas on early ICGA frames (top right) remained non-perfused on late ICGA frames (bottom right) indicating non-perfusion and not only perfusion delay. The pooling on bottom middle late FA frame is explained by ischemia of the outer retina inducing exudation from vessels of inner retina

In 1968 Donald Gass published one more original report in which he described 3 female patients aged 19, 22 and 22 showing rapid loss of central vision associated with multifocal, yellow-white placoid lesions occurring in one eye, with sequential involvement of the second eye, followed by resolution of the fundus lesions and visual improvement over weeks or months thereafter [47]. He called the disease acute posterior multifocal placoid pigment epitheliopathy (APMPPE) and attributed it to the pigment epithelium supposedly responding to a toxic or infectious agent. Not having at his disposition ICGA it is comprehensible that he attributed the disease to the pigment epithelium. However, 4 years later, in 1972 August Deutman, despite limited imaging access to the choroid, as ICGA was not available yet, made the correct interpretation of the lesion process by observing the early frames of FA, showing areas of choriocapillaris non-perfusion [48, 49]. FA did not allow to determine whether these patchy non perfused areas corresponded to absence of perfusion or only perfusion delays. Based on these FA images he called the disease appropriately acute multifocal ischemic choriocapillaritis (AMIC) Very early when ICGA became available it was confirmed that the non-perfused areas seen on early FA frames by Deutman, were indeed not only due to perfusion delay but corresponded to non-perfusion as the choriocapillaris remained non-perfused until late ICGA frames [50]. Gass never admitted Deutman’s explanation of the pathophysiology of the disease and this gave rise each time to polite clashes between the two clinicians when they were both present at meetings. Once they are ingrained in current use, eponyms are very rarely or almost never renamed and APMPPE will remain as the classically accepted denomination of this particular choriocapillaritis (Fig. 4).

### Idiopathic multifocal choroiditis (MFC) (delay in adopting a comprehensive classification)

Multifocal Choroiditis (MFC) belongs to the group choriocapillaritis diseases. It is a bilateral disease usually involving eyes sequentially with recurrences and development of small chorioretinal scars. It affects predominantly young myopic female patients. The mechanism, as in all choriocapillaritis entities, is inflammatory choriocapillaris non-perfusion producing outer retinal ischemia at the origin of the development of inflammatory choroidal neovessels in close to 30% of cases [51].

**Fig. 5:**
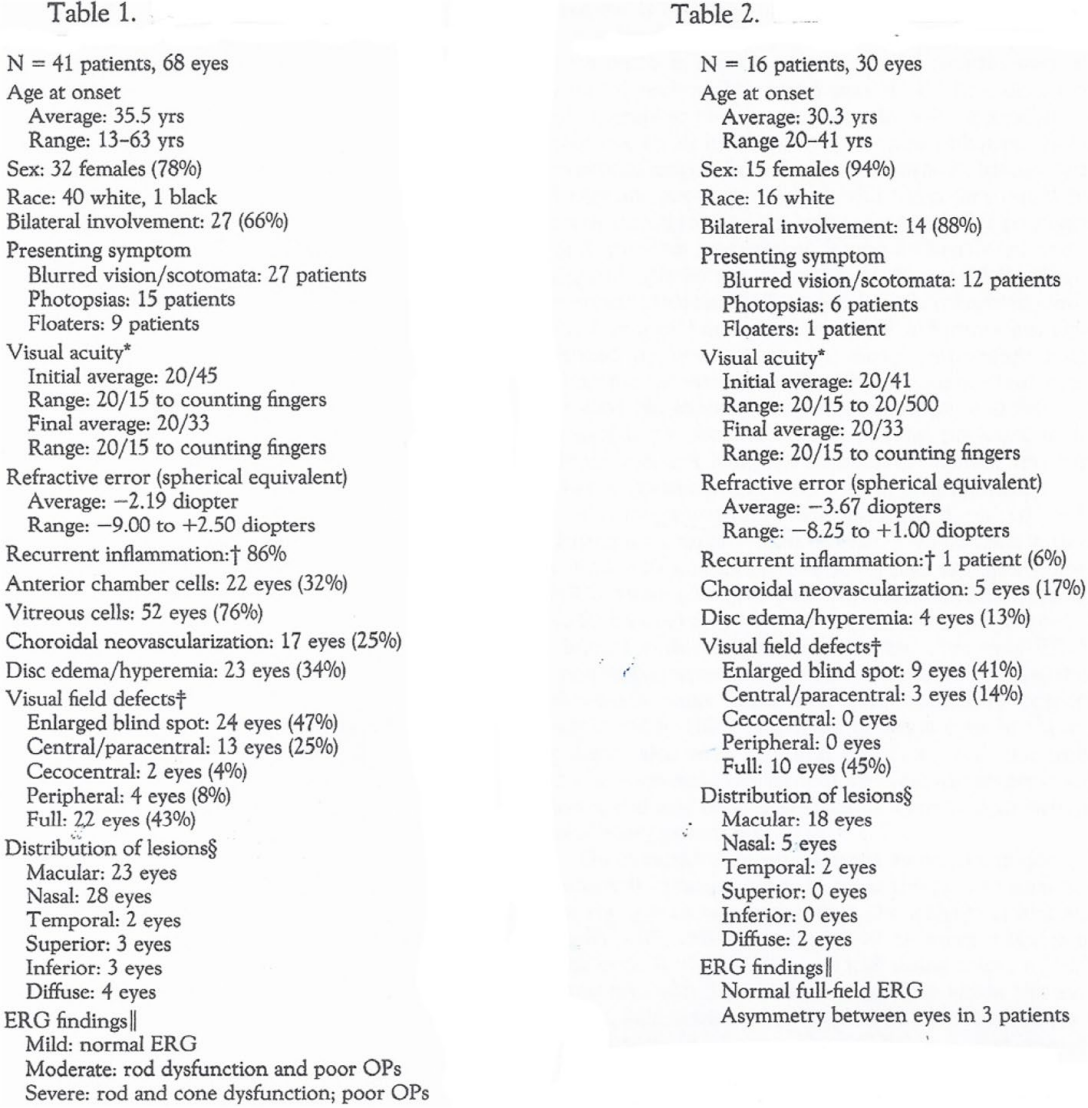
MFC and PIC. Description of MFC and PIC taken from Reddy CV & Colleagues. If the legends concerning both columns are hidden, it is impossible to tell the difference between Tables 1 and 2, clearly indicating that the 2 diseases cannot be distinguished

**Fig. 6:**
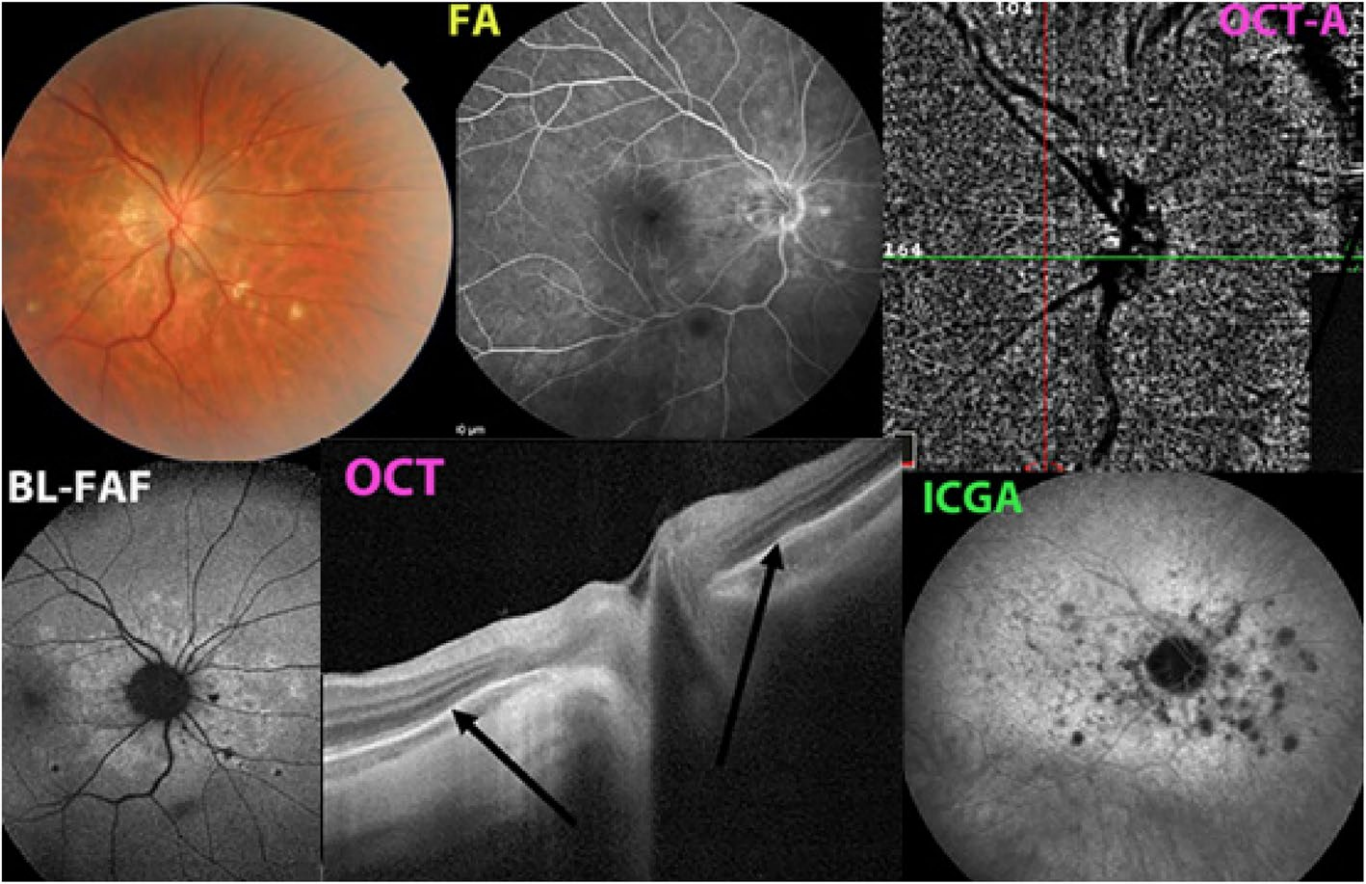
Case of MFC. The disease is characterised by choriocapillaris non-perfusion seen on ICGA as in MEWDS: What distinguishes it from MEWDS is the fact that the disease is recurrent and bilateral and that it shows chorioretinal scars (top left). Non-perfusion is shown on ICGA (bottom right), causing loss of photoreceptor outer segments on OCT (bottom middle) at the origin of hyperautofluorescence (bottom left). The fact that OCT-A seems to show an intact choriocapillaris is explained by the fact that, as in MEWDS, end-choriocapillary vessels are involved which are beyond the reach of OCT-A

In the past there was some confusion of terminology concerning this entity with several denominations duplicating or overlapping MFC, including, among others, Pseudo-POHS looking like presumed histoplasmosis syndrome but negative for histoplasmosis, multifocal choroiditis with panuveitis, an obsolete term as the authors never saw panuveitis in their MFC cases for more than 30 years, and punctate inner choroidopathy (PIC), to cite only a few of the numerous terms around the nebula of MFC. Many of these sub-entities have been lost over the years. In 2013 an editorial put some order in this group of diseases grouping them under the umbrella term of multifocal choroiditis, adding the prefix of idiopathic [52]. Despite this proposed nomenclature, PIC continued to be regarded as a separate entity by some and studies continue to be published relentlessly on PIC or on the characteristics allegedly allowing to differentiate it from MFC. The anecdote that convinced the authors that MFC and PIC were indeed the same disease was an article that listed the different entities [53]. When looking at the characteristics of the two diseases and hiding the legends, we couldn’t say which column corresponded to which disease (Figs. 5 and 6).

### Multiple evanescent white dot syndrome (MEWDS) (inadequate interpretation of signs and inappropriate pathophysiological explanation)

**Fig. 7:**
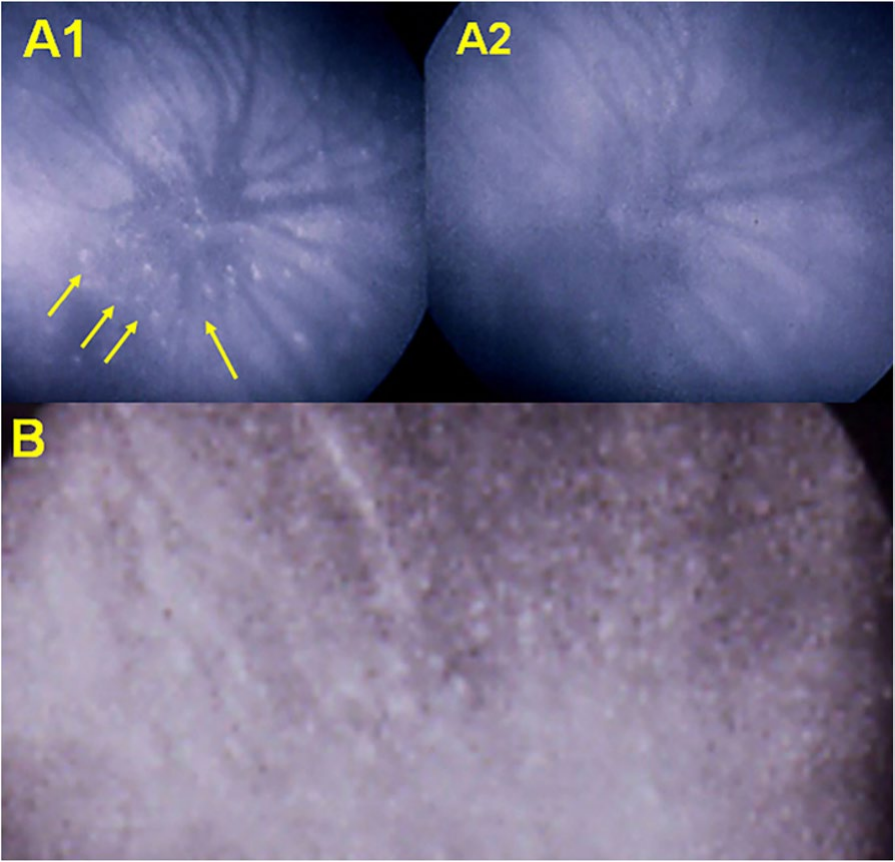
Massive ICG uptake in peripheral fundus lesions of granulomatous chorioretinitis: ICGA peripheral hyperfluorescent pinpoints indicating that diseased cells/tissues, in contrast to what has been published by some, do hyperfix ICG. (A1) hyperfluorescent pinpoints in a tuberculous choroiditis before treatment, resolved after treatment (A2). (B) sarcoidosis choroiditis with numerous ICGA hyperfluorescent pinpoints in peripheral diseased areas. (Reprinted from J Ophthalmic Inflamm Infect. 2021 Dec 18;11(1):45. doi: 10.1186/s12348-021-00279-7. PMID: 34921620; PMCID: PMC8684571)

MEWDS was first described by Lee Jampol and colleagues in 1984 [54, 55]. The subsequent reports were all descriptive reports with lesions thought to involve the photoreceptors and the pigmentary epithelium without however giving pathophysiological explanations on its mechanism [56, 57]. The clinical picture was well defined: unilateral evanescent yellow-white dots of the fundus and mid-periphery, foveal granularity and variable impairment of visual field and visual acuity and recovery within10 weeks. With the availability of ICGA at the beginning of the 1990ties, a first report clearly showed that the primary lesion occurred in the choroid [58]. Numerous reports followed reporting involvement of the choroidal circulation linking it to the other choriocapillaritis entities [59–64]. It was suspected that the choriocapillary non perfusion involved the distal end-choriocapillary vessels explaining why the vascular drop out on ICGA was discreet, non-confluent and reversible. Another argument linking MEWDS to the other choriocapillaritis entities was the large number of reported cases in which MEWDS and other choriocapillaritis entities co-existed or followed each other, clearly speaking for a common nosological mechanism [65–70]. For some time the ophthalmological community was satisfied with the pathophysiological explanation of inflammatory choriocapillaris non-perfusion as the “primum movens”, until OCT-A was added to the imaging armamentarium. OCT-A seemed to show choriocapillaris integrity in MEWDS, so it was logically deduced by some that the primary lesion must be at the level of the photoreceptors [71]. However, it was not paid attention to the fact that there is quasi no flow in end-choriocapillaries involved in MEWDS and flow is needed to be “seen” by OCT-A, as it is the very principle of mechanism of OCT-A. Consequently, OCT-A is unsuitable in this situation being unable to determine whether there is presence or absence of perfusion in these low/no-flow end-choriocapillaries, hence being not an adequate method to analyze this portion of choriocapillaris. In contrast to ICGA, which is probably also able to show hypoperfusion, OCT-A is unable to distinguish different levels of perfusion appearing as normal in mild cases of MEWDS. However, the severity of MEWDS is diverse from one case to another and there are several reports where OCT-A has indeed shown non-perfusion confirming that the pathophysiology is more or less pronounced choriocapillaris non-perfusion that can appear on OCT-A in more severe cases [59, 72–75]. If abstraction is made of the inadequacy of OCT-A for end-choriocapillaris vessels, the alleged integrity of the choriocapillaris in MEWDS could be a seducing theory and it led some to abandon the choriocapillaritis non-perfusion explanation in favor of the hypothesis of primary photoreceptor damage [62]. Finally, there is one big incongruity in the theory of primary involvement of the photoreceptors in MEWDS, namely the explanation of the hypofluorescent areas on ICGA attributed to the hypothetical fact that damaged cells allegedly are not taking up and are no more marked by the ICG molecule. This affirmation was based on in vitro cellular experiments, the same study showing however that damaged cells can have an increased uptake of ICG [76]. In clinical in vivo settings, usually, damaged cells/tissues tend to overfix the ICG molecule and appear hyperfluorescent [77]. This is what is seen in the late phase ICGA in sarcoidosis choroiditis showing peripheral hyperfluorescent pinpoints in affected areas [78] (Fig. 7).

**Fig. 8:**
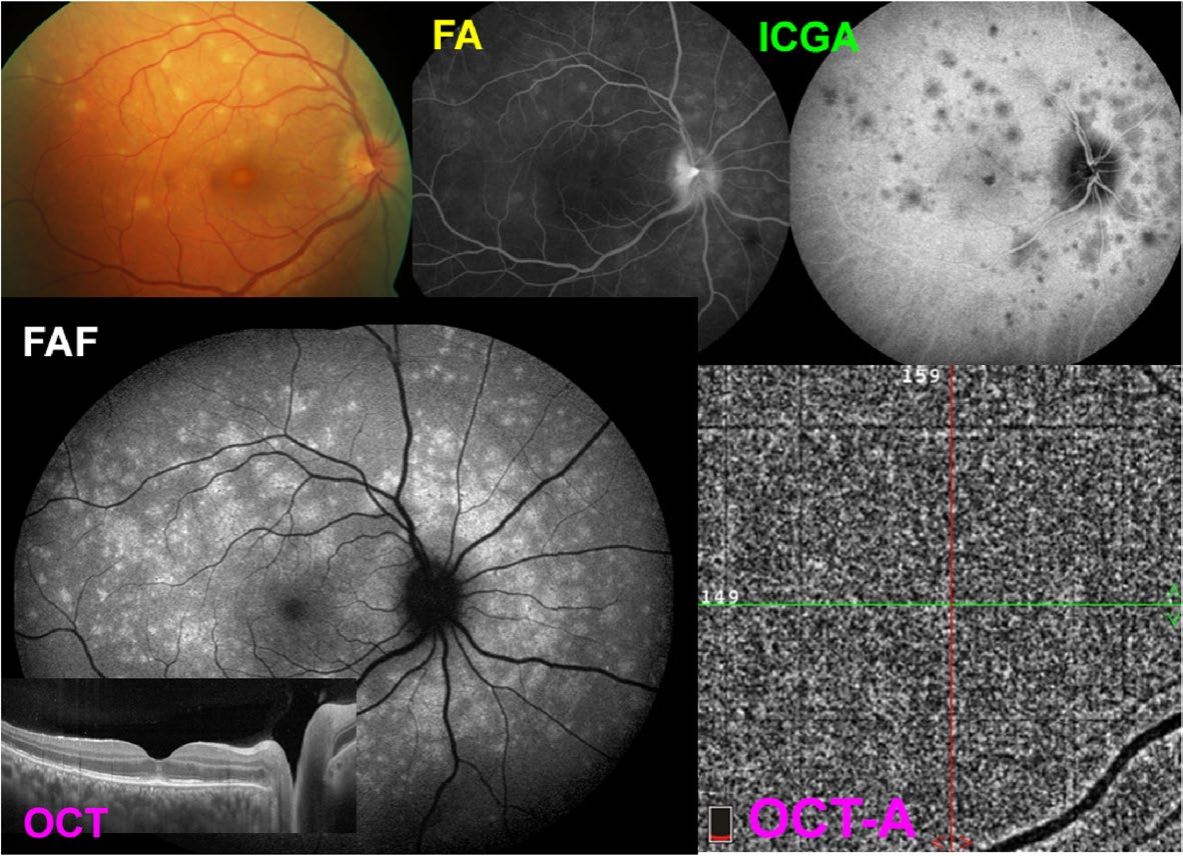
Typical case of MEWDS. MEWDS is explained by end-choriocapillary non-perfusion forming small areas of patchy hypofluorescent spots on ICGA (top right) corresponding to loss of photoreceptor photopigment which causes patchy areas of hyperautofluorescence on FAF (bottom left). Because this level of choriocapillaris is characterized by low or quasi no flow, OCT-A is unable to “see” these vessels and cannot determine whether the vessels are perfused or not. Therefore, on OCT-A, choriocapillaris in MEWDS has erroneously been qualified as normal

Even if the mechanism put forward by the followers of the photoreceptoritis hypothesis were true, during the angiographic phase, ICGA fluorescence is not produced by ICG fixation on cells but by free diffusing of the ICG-protein molecular complex in the choroidal space and can only be explained by reduced or absent perfusion. It is only after the angiographic phase that the very faint remaining ICG fluorescence is explained by tissue fixation or not. The composite Fig. 8 shows an illustration of the modern appraisal of MEWDS, using multimodal imaging (Fig. 8).

The inability of OCT-A to image end-choriocapillary choriocapillaris circulation, the fact that in serious cases of MEWDS drop-outs can appear on OCT-A, the fallacious argument that ICGA hypofluorescent areas allegedly corresponding to “non-uptake” of the ICG molecule by the pigmentary epithelium, the fact that there is a continuum among choriocapillaritis entities with MEWDS being identical to mild early forms of MFC, taken together these arguments clearly point towards a choroidal vascular origin of MEWDS [79, 80].

### Indocyanine green angiography (ICGA) (reluctance to implement its use)

ICGA has been available since the mid-1990ties [81]. It represented a significant leap forward especially for uveitis as it gave precise imaging access to the choroidal compartment quasi-inaccessible for precise investigation by existing modalities. Even though it was clearly a game changer in the appraisal of posterior uveitis, its general implementation was surprisingly slow. Larry Yannuzzi, the early promoter of the technique, noted that the sluggishness of US colleagues to adopt the method routinely certainly played a role in the fact that it was relatively underused. He tried to explain the situation as follows: “Perhaps, the biggest enigma related to ICG angiography is its low utility in the United States compared to the rest of the world. US institutions seldom trained retinal specialists to use ICG angiography. Consequently, the necessary studies to validate the role of ICG angiography for the management of inflammatory and other diseases have never been done” [82]. As a consequence, this disregard of US clinicians had an impact on the global volume of publications and the place of ICGA in the literature was less than what could have been anticipated given the influence of US journals worldwide. This was counteractive for its more generalized implementation and its importance in the mind of clinicians [83].

On the other hand, from the start, European researchers have stressed the essential role of ICGA in choroidal inflammatory diseases for diagnosis and monitoring of such diseases [84]. In choriocapillaritis and stromal choroiditis it is the gold standard as the biomarker for disease activity [85].

When comparing the amount of information, otherwise occult, retrieved by ICGA to the information obtained by FA, the yield is much higher for ICGA for those diseases with choroidal involvement [86]. Indeed, in contrast to deep choroidal lesions, superficial fundus/retinal lesions are not occult and easily accessible to fundus examination. Moreover, superficial fundus structures are more readily accessible to new non-invasive imaging methods such as OCT or OCT-A, which is hardly the case for the choroid with a considerable “return on investment” for ICGA. Despite these objective advantages, it is a fact that ICGA has been underused for diverse reasons including the fact that it is an invasive procedure, clinicians are not familiar with the procedure, its protocol and/or its interpretation, and because of the cost of the ICG dye.

Although the yield from ICGA is much higher than from FA in chorioretinitis [86], in clinical practice, the latter is routinely performed whereas ICGA is often neglected and not deemed necessary. Very often do we get referrals where FA has been performed as a routine for which angiography has to be repeated to have ICGA results in order to have available all the global information on posterior uveitis.

**Fig. 9:**
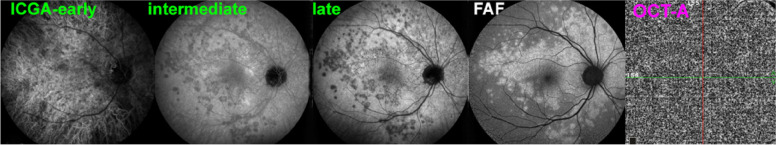
Typical case of MEWDS. MEWDS is the consequence choriocapillaris circulation anomaly. In the very early ICGA frame (left picture) there is perfusion delay. Second from left frame shows the intermediate angiographic phase with small areas of end-choriocapillary non-perfusion becoming to be apparent against a fluorescent background. The middle frame shows more clearly the areas of non-perfusion as the contrast towards the fluorescing background is more pronounced. The second picture from the right shows intense fundus autofluorescence indicating loss of photopigment. The last picture to the right is an OCT-A picture showing an apparently normal choriocapillaris, being unable to image end-choriocapillaries

More recently the tendency towards decreased use of ICGA has been reinforced by the belief that multimodal imaging, especially OCT-A, could replace ICGA [87]. This is implicitly hinted at in many publications on OCT-A [88–90] without however having been yet explicitly asserted [91]. Many publications using multimodal imaging, even for research purposes, do not bother to include ICGA any longer. However, in many pathologies involving the choroid, ICGA is essential to comprehensively analyze the pathophysiological mechanisms as explained for MEWDS in the previous section and demonstrated again in Fig. 9. For instance, in the case of MEWDS, only ICGA can prove that the disease process is choroidal vascular, namely end-capillary choriocapillaris non-perfusion.

**Fig. 10:**
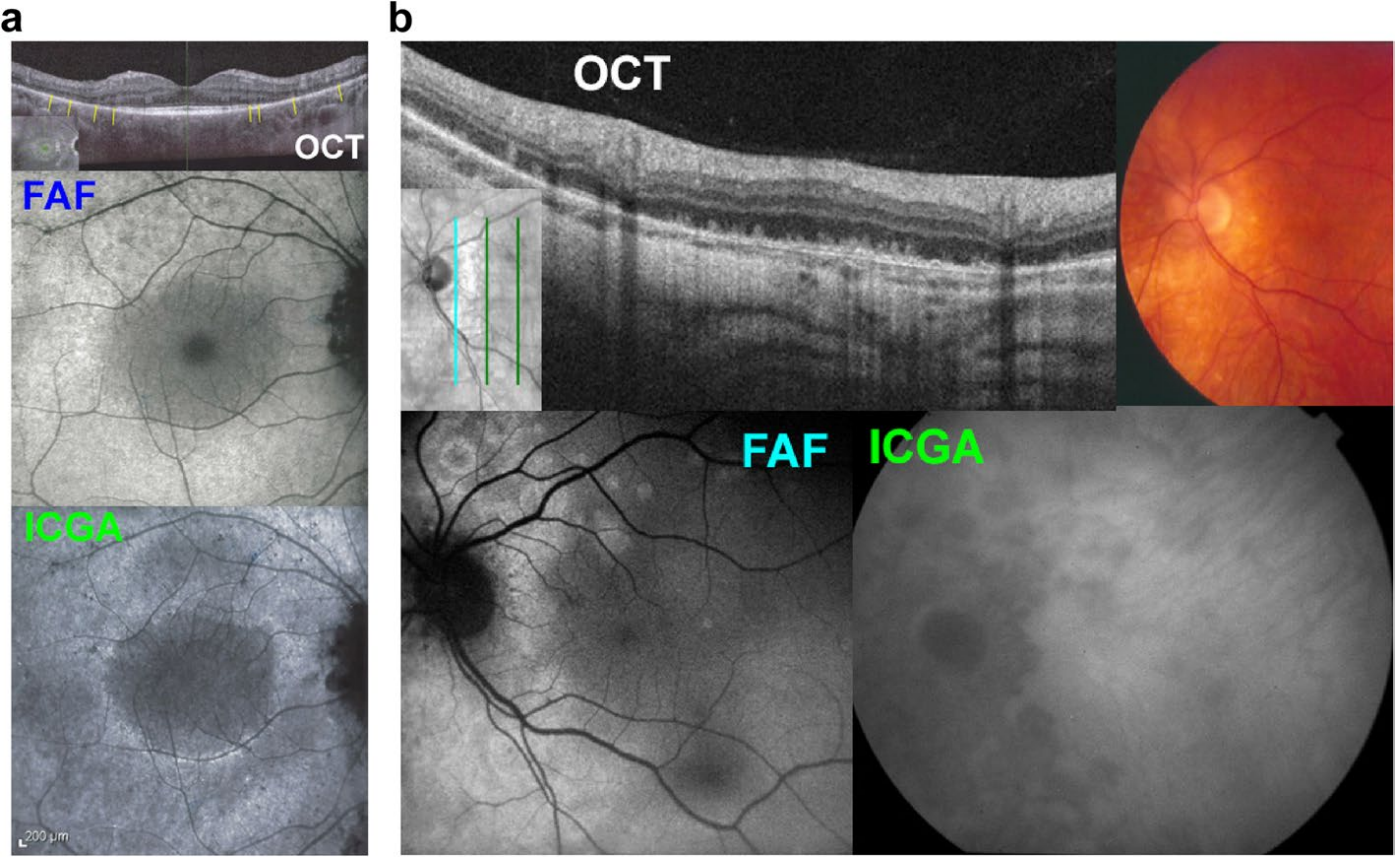
**a** & **b** ICGA is essential in differentiating photoreceptoritis from outer retinal ischemic damage to photoreceptors due to choriocapillaritis. Loss of photoreceptors produces hyperautofluorescence whether due to direct insult to photoreceptors or secondary to ischemia due to choriocapillaritis. ICGA is necessary to differentiate these two mechanisms. **a** Loss of photoreceptors due to direct, primary lesion to the photoreceptors in a case of AZOOR. Top picture, OCT showing loss of photoreceptors except in the central area producing diffuse hyperautofluorescence except in the central area where photoreceptors are conserved. The bottom ICGA picture shows absence of non-perfusion indicating integrity of choriocapillaris. Central more dark area is due remaining photopigment. **b** Choriocapillaritis induced secondary loss of photoreceptors in a case of idiopathic multifocal choroiditis. Fundus picture (top right) showing faint chorioretinal scars. Massive loss of photoreceptors on OCT (top left, insert shows localization of scan) with corresponding areas of hyperautofluorescence on FAF (bottom left), corresponding to an area of non-perfusion on the ICGA frame (bottom right)

Further, ICGA is indispensable and should be part of a multimodal analysis to determine whether outer retina diseases are due to direct photoreceptor damage as in acute zonal outer occult retinopathy (AZOOR) or are the result of secondary damage due to ischemia produced by choriocapillaris non-perfusion [92]. On Fig. 10, multimodal imaging shows primary photoreceptor disease in a case of AZOOR (Fig. 10a) and secondary photoreceptor disease in a case of MFC. ICGA is needed to show that choriocapillaris perfusion is conserved in AZOOR indicating primary photoreceptor disease, whereas in the case of MFC there is choriocapillaris non-perfusion indicating that loss of photoreceptors is secondary to choriocapillaritis induced ischemia (Fig. 10b).

### The inappropriate term of cyanescence in ICGA (misnomer)

Together with ICGA, appeared a few years later the term of cyanescence used either to speak of hypocyanescence or hypercyanescence. When searching for the term of cyanescence in the dictionary there is no entry. Cyan is the name of the blue-green color, but cyanescence has no meaning.

The phenomenon of fluorescence is described as the property of some atoms and molecules to absorb light at a particular wavelength and to subsequently emit fluorescing light of a longer wavelength after a brief interval. The molecule of indocyanine green has the property of fluorescence as it emits light at the 830 nm wavelength while absorbing light at the 800 nm wavelength [93]. Fluorescence takes its name from one of the fluorescing molecules, namely fluorescein sodium. At one moment one clinician or several decided to call ICGA fluorescence cyanescence speaking of hyper or hypocyanescence by analogy to the terms hyper or hypofluorscence used for FA. The physical phenomenon occurring in ICGA is indeed fluorescence and it should be called such, while cyanescence is a non-existent term with no meaning, an invented misnomer. Indeed, in the first articles, when it was used, it was always attached to fluorescence, “fluorescence/cyanescence” so that the reader could understand what was really meant by cyanescence was fluorescence [94], hence a meaningless term as it does not represent the fluorescence property of ICG. In the following articles it was then used independently. As indicated, cyan is the ancient Greek word for turquoise with a predominant wavelength between 500 and 520 nm nowhere close to the emission of indocyanine green. A quick search on PubMed only for the term hypocyanescence showed 46 items with use of the inappropriate term in many centers all around the world [95]. It is always puzzling to note how quickly new pseudo-scientific terms are taken up by clinicians without questioning their accuracy.

## Discussion

The few inadequacies, among others, discussed in our article are due to several very diverse factors, each one having its peculiarities. One striking feature is the lack of critical sense of the medical community in the endorsement of new terminologies, concepts and guidelines and the inertia to revise erroneous precepts, as they are passed on and on without questioning. A corollary to this propensity is the excessive often misused influence of opinion leaders whose proposals are often accepted without questioning, especially when they are published in high impact journals. The “white dot syndromes” case is perfectly illustrating this phenomenon. It is still difficult to understand how such an unfounded concept imposed itself like a bushfire and was accepted without questioning in a record time by all the most respected instances of ophthalmology. In this case the concept was proposed by two opinion leaders. However, what classically occurs is the establishment of large, if possible international groups around a novel notion to ensure its acceptance. This is what occurred with the concept of MEWDS being presented as a primary photoreceptor disease, making it difficult to go against an imposing group of high-grade specialists.

Another origin of potential inadequacies lies in the process of consensus meetings. Such meetings are sometimes called by influential concerned groups aiming at redefining a disease or a group of diseases. It is sometimes not impossible that pre-determined possibly biased concepts get adopted by an international floor resulting in inadequate guidelines which truly was the case for BRC and VKH as indicated hereabove. The flaw in such a process is that it does not consider divergent views [96]. For instance, guidelines enacted by a respected, undisputable and worldwide acclaimed group of specialists in nomenclature may still represent one part of the reality, making it however difficult to have an audible divergent voice.

Another explanation of inadequacies is the lack of investigational means available at the time of the description of a disease, which was the case when Gass described APMPPE, only able to rely on fundus examination and fluorescein angiography, favouring the hypothesis of a primary retinal pigment epithelial disease. Nevertheless, Deutman with same investigational modalities available was able to describe the correct mechanism of APMPPE, ischemic choriocapillaritis.

Slow-wittedness, hierarchy and conservatism explain the resistance towards adopting the comprehensive classification (MFC) and the perpetuation of the often inappropriate *no-treatment” policy of APMPPE repeated from one textbook to another.

The propensity of opinion leaders to invent whimsical terminologies is at the origin of definitions difficult to understand rather than to privilege obvious straightforward self-explanatory descriptions, less flabbergasting but easy to understand by all clinicians. Cyanescence is such an occurrence as well as the term bacillary layer detachment and others that we will appraise in our next report. Finally in the case of zoster, stubbornness and abuse of influence of persons in a leading institution recommending not to follow obvious treatment improvements deprived patients from a reimbursed efficacious treatment for several years.

These diverse mechanisms leading to inadequacies may have serious consequences on the knowledge and/or practice of medicine and may be difficult to be updated.

## Data Availability

No datasets were generated or analysed during the current study.

## Abbreviations

ICGA: Indocyanine green angiography
FA: Fluorescein Angiography
APMPPE: Acute posterior multifocal pigment epitheliopathy
WDS: White dot syndromes
BRC: Birdshot retino-choroiditis
VKH: Vogt-Koyanagi-Harada
MFC: Multifocal choroiditis
AHZO: Acute herpes zoster ophthalmicus
ACV: Acyclovir
HDDs: Hypofluorescent dark dots
KPs: Keratic precipitates
SUN: Standardization of Uveitis Nomenclature
MEWDS: Multiple evanescent white dots syndrome
IUSG: International Uveitis Study Group
MMI: Multimodal imaging
SO: Sympathetic Ophthalmia
SC: Serpiginous choroiditis
BCVA: Best corrected visual acuity
RPE: Retinal pigment epithelium
OCT-A: Optical coherence tomography-angiography
SD-OCT: Spectral Domain Optical Coherence Tomography
AMIC: Acute multifocal ischemic choriocapillaritis
PIC: Punctate inner choroidopathy
AZOOR: Acute zonal outer occult retinopathy
